# Association between Children’s Difficulties, Parent-Child Sleep, Parental Control, and Children’s Screen Time: A Cross-Sectional Study in Japan

**DOI:** 10.3390/pediatric15040060

**Published:** 2023-11-08

**Authors:** Yusuke Arai, Daimei Sasayama, Kazuhiro Suzuki, Toshinori Nakamura, Yuta Kuraishi, Shinsuke Washizuka

**Affiliations:** 1 Department of Psychiatry, Shinshu University School of Medicine, Matsumoto-City 390-8621, Japan; y-arai@shinshu-u.ac.jp (Y.A.); toshinaka@shinshu-u.ac.jp (T.N.); y-kuraishi@shinshu-u.ac.jp (Y.K.); swashi@shinshu-u.ac.jp (S.W.); 2 Department of Psychiatry, Kurita Hospital, Nagano-City 380-0921, Japan; 3 Department of Community Mental Health, Shinshu University School of Medicine, Matsumoto-City 390-8621, Japan

**Keywords:** screen time, child psychiatry, sleep, parent-child relation

## Abstract

Children’s screen time may affect their growth and development. However, differences in the impact of various psychiatric and psychological factors on children’s screen time is a research gap. This study aimed to explore the differences in the influence of related factors affecting children’s screen time based on their sleep, difficulties, and parental control among Japanese elementary and junior high school students. A cross-sectional survey was conducted among parents in Japan. Data on screen time duration, parent–child background, strengths and difficulties, sleep variables, and parental control types were collected from 225 households. A regression analysis revealed that high Strengths and Difficulties Questionnaire (SDQ) scores (β = 0.166, *p* = 0.008), sleep duration (β = −0.281, *p* < 0.001), and parental control (β = −0.204, *p* = 0.001) were significantly related to children’s screen time. Additionally, it was found that parents’ late bedtimes affect children’s screen time by mediating children’s sleep duration. This study, together with previous research, provides comprehensive insights into design interventions to decrease the screen time of children in the Japanese context.

## 1. Introduction

Since 2005, with the proliferation of digital devices, there has been an exponential increase in screen time studies. After the coronavirus pandemic in 2019, there was an increase in children’s sedentary and screen time [1], and many studies explored the potential negative effects on children’s health. In Japan, the Ministry of Education, Culture, Sports, Science, and Technology introduced the “GIGA School Initiative” [2] in December 2019, aiming to provide every elementary and junior high school student with a digital device for effective learning at school and home. As digital devices swiftly integrate into the daily lives of Japanese children, concerns regarding the negative physical and developmental effects of screen time are growing, drawing the attention of educators, medical professionals, and parents alike. Some Japanese elementary and junior high schools have implemented initiatives to restrict screen time for their students. These efforts involve collaboration between schools and parents and continue for ten days to manage students’ screen time. However, relying solely on these measures may not be sufficient to ensure a long-term reduction in screen time. Overseas studies have suggested that, in addition to such interventions, education on the health consequences of screen time for teachers and children and frequent distribution of newsletters to parents can effectively reduce screen time [3,4]. The Japanese Pediatric Society recommends limiting screen time to two hours or less per day for children aged two years and above [5]. This recommendation is based on previous research indicating the risks of excessive screen time, such as obesity [6], sleep disorders [7], emotional and behavioral problems [8], impaired vision [9], and academic underachievement [10]. Factors contributing to excessive screen time include child difficulties such as hyperactivity/inattention, emotional symptoms, conduct problems [11], and short sleep duration [12]. In terms of neuropsychiatric and psychological effects, a 2018 review by Lissak G. [13] indicates that excessive screen time correlates with sleep deprivation, depressive symptoms, behaviors related to Attention-Deficit/Hyperactivity Disorder (ADHD), diminished social coping abilities, and craving behaviors like substance dependence.

Recent studies on screen time and sleep have found significant correlations. Extended screen time is linked with decreased sleep duration, prolonged sleep onset latency, excessive daytime sleepiness, insomnia, and heightened severity of overall sleep disorder symptoms [7]. This relationship between screen time duration and sleep quality and duration is observed from infancy to adolescence [14]. Longitudinal studies suggest that insufficient sleep can lead to fatigue and subsequent drowsiness the following day, which may increase screen time and sedentary behavior [15]. In conclusion, a bidirectional relationship exists between prolonged screen time and poor sleep, and these concerns are applicable across childhood.

Much debate exists regarding the causal relationship between neurodevelopmental disorders and screen time. ADHD is characterized by heightened attention and impulsivity. Recent research indicates a connection between ADHD-related symptoms and excessive screen time. Children diagnosed with ADHD, or those assessed for attention and impulsivity problems, tend to have more screen time than their peers without these diagnoses [8]. A large-scale cross-sectional study revealed a correlation between screen time and emotional and behavioral issues, including ADHD and adverse life events [16].

Furthermore, a 13-month longitudinal study found that increased screen time activities, like playing video games or watching TV, correlated with a rise in future attention-related problems [17]. These results suggest an interactive relationship between ADHD-related symptoms and excessive screen time. Similarly, the causal relationship between screen time and Autism Spectrum Disorder (ASD) has been debated for some time. A recent longitudinal study suggests that increased screen time might be an early indicator of ASD rather than a result of an individual’s genetic predisposition to the disorder [18].

Sleep disturbances are commonly observed in children and adolescents with ASD and ADHD. A recent meta-analysis investigating the prevalence of sleep disturbances in ASD found an overall rate of 13% within the ASD population. This starkly contrasts with the prevalence of 3.7% in the general population [19]. Regarding ADHD, a recent cohort study reported that 7.5% of individuals with the disorder were diagnosed with sleep disturbances, with 47.5% being prescribed sleep medications [20]. Furthermore, a study has indicated a weak negative correlation between the Children’s Strengths and Difficulties Questionnaire (SDQ) scores and sleep duration [21]. These findings underscore a potential connection between sleep disorders and symptoms typical of neurodevelopmental disorders.

Parent–child dynamics have been demonstrated to influence the duration of screen time. Recent research suggests a positive correlation between prolonged screen time and family conflicts [22]. Furthermore, a child’s perception of their parents’ involvement is inversely related to screen time before bedtime [23]. It is also noteworthy that parental controls are associated with children’s TV viewing patterns. Specifically, a parent’s confidence in limiting viewing time has an independent effect on a child’s TV watching during weekdays, acting as a bridge between parental control and the overall screen time duration [24]. There are limited studies on the relationship between parental sleep and children’s sleep duration. However, evidence suggests a link between a parent’s late bedtime and their child’s sleep problems [25].

In summary, from psychiatric and psychological perspectives, a clear association exists between screen time and various factors, including symptoms characteristic of children’s neurodevelopmental disorders, sleep duration, and parent–child dynamics. However, we recognize as a research gap that there are limited studies about differences in the impact of various psychiatric and psychological factors on children’s screen time and their interconnectedness within a uniform population. This becomes especially crucial when devising national strategies to reduce screen time, as the influence of each factor on screen time likely varies with the cultural context of individual countries. Given this backdrop and considering the rapid progression of the “GIGA School Initiative” in Japan, we found it imperative to address this research void and thus structured our study accordingly.

The primary objective of this study was to examine the differential impacts of psychiatric and psychological factors on children’s screen time among Japanese elementary and junior high school students. Specifically, we investigated factors such as children’s sleep duration, SDQ scores, and the degree of parental control. A secondary objective was to explore the interrelations among these factors. Given the established link between parents’ late bedtimes and children’s sleep disorders [25], we hypothesized that children of parents with later bedtimes would experience reduced sleep and increased screen time. This hypothesis was examined using mediation analysis. Finally, by synthesizing the findings of this study with prior research, we propose specific interventions tailored to reduce children’s screen time within the Japanese context.

## 2. Materials and Methods

### 2.1. Ethics Statement

This study’s protocol was approved by the institutional review board of Shinshu University (approval number 5926). This study was conducted by a school nurse who obtained permission from the principals of the participating schools. The questionnaire was administered to the parents of the students. Shinshu University collected the data, ensuring the privacy and confidentiality of the participants’ personal information. Then, the university conducted statistical analyses using the de-identified data. These measures were taken to protect the privacy and confidentiality of the participants’ data in accordance with ethical guidelines.

### 2.2. Study Design and Participants

This multi-center cross-sectional survey was conducted from 17–26 June 2023 and targeted 438 households in Nagano Prefecture, Japan. We focused on parents and children from elementary and junior high schools in the northern part of Nagano Prefecture. To qualify for the survey, participants had to be parents. Only one member from each household was surveyed. If a participant had multiple children, they were instructed to provide answers about their oldest child, who was in grade nine.

Given that the survey aimed to capture data from typical households, we did not set any exclusion criteria based on the background factors of the children or caregivers. In each household, a school nurse approached the primary caregiver of an elementary or junior high school student and requested them to complete the survey using Google Forms. A total of 225 (51.4%) households participated in the survey. Given the exploratory nature of this study, we did not conduct any a priori sample size calculations.

### 2.3. Procedures and Measures

The background information was collected from the questionnaire survey, which included the students’ school year, sex, number of siblings, age, and sex of the primary caregiver. For the assessment of screen time, participants were requested to report children’s average daily usage time (excluding the usage to study), including weekends, over the past week. The screen time durations were recorded in 30 min increments, ranging from 0 to 300 min. Regarding sleep-related variables, students’ habitual bedtime and wake-up time, as well as their parents’ bedtime and wake-up time on weekdays, were investigated. Based on these variables, the time in bed (TIB) was calculated. Additionally, the parents were administered the SDQ [26] to assess the child’s difficulties. The total and specific sub-domain scores were calculated. Regarding parental control of screen time, the survey was conducted using the following: (1) No rules, (2) Promise without compliance, (3) Verbal promise with compliance, (4) Automated time limiting.

### 2.4. Statistical Analysis

Continuous variables were summarized in descriptive statistics using means and standard deviations, while categorical variables were presented as frequencies and proportions. Appropriate statistical methods such as the Kruskal–Wallis test and Spearman’s correlation analysis were employed to analyze the relationships between main model variables. The significance level for the Kruskal–Wallis test was adjusted to two-tailed *p* < 0.05. The relationships between model variables were analyzed as follows. The mean screen time for each group based on the SDQ scores (low-need: 0–13, some-need: 14–16, high-need: 17–40) was compared using the Kruskal–Wallis test. A principal component analysis for sleep variables was performed due to potential correlations between the school year and sleep variables (TIB and bedtime). The extracted principal component, “sleep duration”, was evaluated for its correlation with screen time using Spearman’s correlation analysis. The correlation between parental and child bedtime was investigated using Spearman’s correlation analysis. Screen time between parental control sub-groups was compared by categorizing parental control into three levels based on the survey results: “No control” = no rules or promise without compliance, “Verbal promise” = verbal promise with compliance, and “Automated time limiting”. We employed Spearman’s correlation analysis to examine the relationships between each factor that was used as an explanatory variable in the multiple regression analysis. After verifying the absence of significant correlations between these factors (Table 1), we proceeded with a stepwise multiple linear regression analysis to assess the impact of each factor on screen time. The hypothesis that parents’ later bedtimes would be associated with less sleep and increased screen time for their children was tested using a path analysis. Path analysis was performed using the Statistical Package for the Social Sciences Amos version 29 (SPSS; IBM Corp., Armonk, NY, USA), while other analyses were conducted using SPSS Statistics version 29 (IBM Corp., Armonk, NY, USA).

## 3. Results

### 3.1. Demographic and Clinical Characteristics

Table 2 presents the demographic and clinical characteristics of the children and caregivers. The children’s mean (standard deviation, SD) screen time was 133.6 (67.37) minutes. The average grade of the surveyed participants was 6.16 (2.52), and 82.7% were above fourth grade. The children’s average bedtime and wake-up time were 22:03 (56 min) and 6:29 (31 min). For students in grades 4–6, the average bedtime and wake-up time were 21:42 (31 min) and 6:32 (22 min), respectively; for junior high school students, they were 22:30 (53 min) and 6:32 (36 min), respectively. Students in grades 4–6 and junior high school tended to have earlier bedtimes and wake-up times than those mentioned in the *Children and Youth White Paper* for the fiscal year 2015 [27]. According to the National Living Time Survey, the average TIB for primary caregivers was 6 h 54 min, which is equivalent to 6 h 53 min for women in their 40s [28]. The average total score of the SDQ was 9.71 (5.21). Among the children, 26 (11.7%) met the criteria for “High need”, while 23 (10.2%) met the criteria for “Some need”.

### 3.2. Association between Screen Time and SDQ, Sleep Variables, and Parental Control

**Figure 1:**
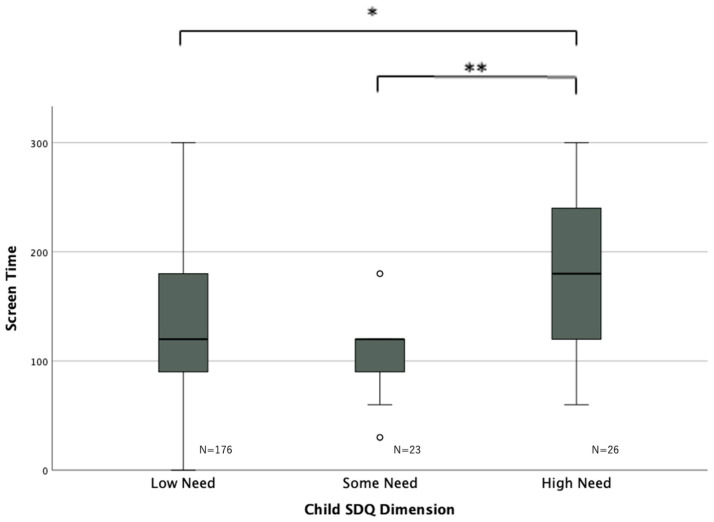
Association between screen time and SDQ score. * *p* < 0.05, ** *p* < 0.001, ○: outlier. There were significant variations in screen time between the three groups, with the high-need group showing higher screen time than the some-need and low-need groups. Abbreviations: SDQ = Strengths and Difficulties Questionnaire.

The mean screen time for the three groups based on SDQ scores was compared (Figure 1) using the Kruskal–Wallis test. Significant differences were found between the high-need and some-need groups (high-need–some-need (standard error): 58.42 (18.40), *p* = 0.004), as well as between the high-need and low-need groups (high-need–low-need (standard error): 35.76 (13.50), *p* = 0.024).

Factor analysis of sleep variables was conducted to account for each sleep variable and school year. The sample adequacy measure (Kaiser–Meyer–Olkin) was 0.693, indicating sufficient suitability for the analysis. The factor matrix revealed that the first principal component was strongly correlated with TIB (correlation coefficient of 0.919) and was named the “sleep duration” factor. There was a weak negative correlation between “sleep duration” and screen time, with a Spearman’s rank correlation coefficient of −0.225 (95% confidence interval, CI: −0.349 to −0.093, *p* < 0.001). Additionally, the correlation coefficient between parental and children’s bedtimes was 0.368 (95% CI = 0.246–0.479, *p* < 0.001).

**Figure 2:**
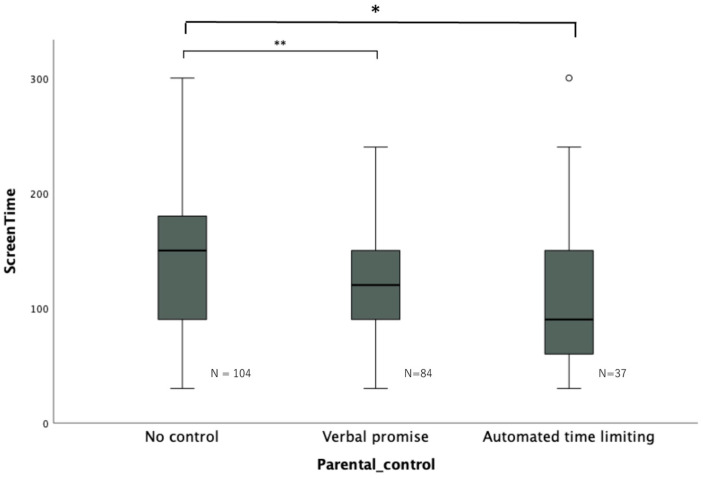
Association between screen time and parental control level. * *p* < 0.05, ** *p* < 0.01, ○: outlier. There was a significant difference in screen time between groups, with “Automated time limiting” and “Verbal promise” having shorter screen time than “No control”.

Figure 2 presents the results of the Kruskal–Wallis test, which assessed the differences in average screen time among the three sub-groups based on parental control levels. The findings indicated that both the “Automated time limiting” and “Verbal promise” groups had significantly shorter screen time compared to the “No control” group. Specifically, the screen time was significantly shorter in the “Automated time limiting” group compared to that in the “No control” group (Automated time limiting–No control (standard error): −35.52 min (12.30), *p* = 0.012). Similarly, the screen time was significantly shorter in the “Verbal promise” group compared to the “No control” group (Verbal promise–No control (standard error): −34.00 min (9.43), *p* = 0.001).

### 3.3. Stepwise Multiple Linear Regression Analysis for Children’s Screen Time

The stepwise multiple linear regression analysis results indicated that the model accounted for 14.2% of the variance in children’s screen time (R^2^ = 0.154, adjusted R^2^ = 0.142). Influential factors for children’s screen time included children’s difficulty (β = 0.166, *p* = 0.008), children’s sleep duration (β = −0.281, *p* < 0.001), and parental control (β = −0.204, *p* = 0.001), as presented in Table 3.

### 3.4. Indirect Effect of Parental Bedtime on Children’s Screen Time

**Figure 3:**
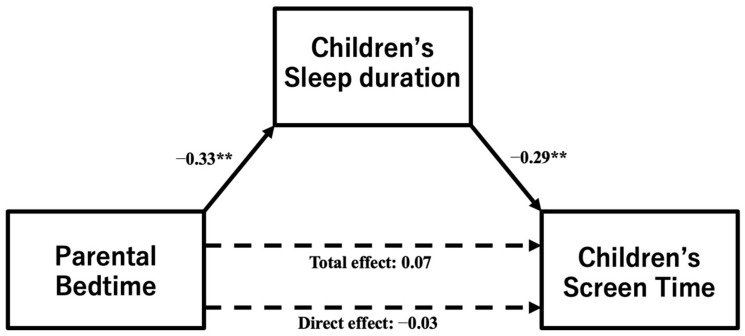
A path analysis to test the hypothesis that parents’ later bedtimes would be associated with less sleep and increased screen time for their children. ** *p* < 0.001.

A path analysis indicated that parental bedtime had a significant negative correlation with children’s sleep duration (β = −0.325, *p* < 0.001) (Figure 3). This suggests that when parents go to bed later, their children’s sleep duration becomes shorter. While there was no significant direct or total effect of parental bedtime on children’s screen time (direct effect; β = −0.027, *p* = 0.689, total effect; β = 0.068, *p* = 0.308, as seen in Figure 3), the indirect effect was significant, as detailed in Table 4 (β = −0.095, *p* = 0.001). This supports the hypothesis that parents’ later bedtimes influence children’s sleep duration and indirectly increase their screen time.

## 4. Discussion

This study found that the high difficulties assessed by the SDQ and short sleep duration are associated with increased screen time among Japanese elementary and junior high school students. These findings are consistent with previous research [29,30,31,32,33,34,35,36,37] and might be generalizable in the Japanese context. Furthermore, the findings indicated that parental control is related to reduced screen time among elementary and junior high school students in Japan. These results are significant considering the limited research on the effectiveness of parental control in the Japanese context. Specifically, “Automated time limiting” and “Verbal promise” were highly related to screen time reduction.

Furthermore, the impacts of children’s sleep duration and parental control on screen time were higher than children’s difficulty. In summary, for Japanese elementary and junior high school students, extending children’s sleep duration and strengthening parental control may be more effective in reducing screen time than reducing children’s difficulty. Although the total effect was not significant, considering the significant indirect effect of parental bedtime on screen time via children’s sleep duration, it is suggested that bringing forward parental bedtime is important for reducing screen time. These results are encouraging information from the perspective of clinical psychiatry and school health guidance. This is because intervention for children’s difficulty requires a high level of expertise, and the number of experts is limited, whereas providing parents with information about parental bedtime and parental control is a common practice in the psychiatric and school health field.

In summary, as future measures to reduce screen time in Japan, bringing forward parental bedtime and providing parental control guidance to parents can have a high impact and can be generalized. Furthermore, regarding children’s sleep duration, which had the most impact on screen time in this study, a national campaign for adolescents called “early to bed, early to rise, and do not forget your breakfast” has already been implemented in Japan. This study supports this measure, especially “early to bed”.

The squared multiple correlation coefficient for a screen time regression analysis of 0.142 indicates a weak to moderate effect, so it is important to consider other factors that may influence screen time. Since this study was only evaluated from a psychiatric and psychological perspective, it is important to consider factors related to the environment, body, and habitual behavior. Previous studies have shown that having a television in the bedroom [38], insufficient physical activity [39], the number of devices owned by a child [40], and parental screen time [40] are associated with lower screen time. Future research should develop and test hypothetical models that include these factors.

Based on these results and previous findings, we would like to discuss ways to reduce screen time through a collaborative program between parents, schools, and mental health professionals that is feasible and effective in Japan. According to previous research, educating children about screen time at school, providing regular newsletters to parents, educating teachers, and implementing a screen media time reduction challenge of around ten days effectively reduced children’s screen time [3,4]. Based on these findings, newsletters and school-based educational initiatives appear promising tools for educating parents and children. For these interventions to be truly impactful, it is crucial to outline effective and implementable content in them. Reinforcing this, a recent Japanese cohort study reported that only approximately 5–10% of children across all grades and genders adhere to the “early to bed, early to rise, and breakfast” routine [41]. Considering our research findings, it is important to include information that advancing parental bedtime can lead to an increase in children’s sleep duration and a reduction in screen time in newsletters and school education.

In summary, it is important for parents and children to practice “early to bed, early to rise, and do not forget breakfast”. In addition, incorporating specific instructions on utilizing the automatic time limit feature available on certain digital devices could enhance the newsletter’s efficacy. For devices distributed by the school, presetting this time limit feature would be prudent. While implementing a population-based approach is of utmost importance, screening children’s difficulties and facilitating their connection to child psychiatrists is also important. Specifically, it may be effective to reduce screen time if school nurses regularly conduct SDQs to understand children’s difficulties and proactively connect high-risk children with child psychiatrists. It would also be possible to incorporate the SDQ as a questionnaire for parents during the 10-day screen media time reduction challenge.

This study had some limitations. First, its applicability to a broader context may be limited due to its focus on specific regions within Japan. Second, data were collected exclusively through surveys, introducing the possibility of parental subjectivity and memory biases. This underscores the need for large-scale and objective research in the future. Third, the small sample size limited the generalizability of the findings. As this was an exploratory study, the sample size was determined from available data without prior power analysis. Fourth, although this study targeted elementary and junior high school students, 82.7% were in grade four or higher. This concentration inherently limits the generalizability of our findings to the entire age group. Finally, it is essential to highlight that no definitive causal relationship was established in this study. Moreover, the enduring consequences of heightened parental control remain uncertain.

## 5. Conclusions

This study revealed that children’s difficulties and shorter sleep duration are associated with increased screen time, while more parental control is related to reduced screen time in the Japanese context. Furthermore, we found that parental later bedtimes not only affect children’s sleep duration but also indirectly increase children’s screen time. To strengthen the validity of these results, it is crucial to conduct large-scale and objective research, as well as to explore factors that were not investigated in this study. Thus, we can gain a more comprehensive understanding of the complex factors influencing screen time in Japanese elementary and junior high school students.

## Figures and Tables

**Table 1:** Spearman’s correlation analysis to examine the relationships between each factor.

Variables	Spearman’s Rho	*p*	95% CI	
			Lower	Upper
Children’s difficulty–Children’s sleep duration	0.114	0.088	−0.021	0.245
Children’s difficulty–Parental control	−0.061	0.365	−0.194	0.075
Children’s sleep duration–Parental control	0.086	0.2	−0.049	0.218

Notes: Abbreviations: CI = confidence interval.

**Table 2:** Demographic and clinical characteristics.

N = 225	
Background	Mean (SD or %)
School year	6.16 (2.52)
Sex male	110 (48.9%)
Siblings	2.08 (0.76)
Age (primary caregiver)	44.0 (6.13)
Sex male (primary caregiver)	26 (11.6%)
Screen time	Mean (SD)
All students (N = 225)	133.60 (67.7)
School year 1–3 (N = 39)	120.00 (57.6)
School year 4–6 (N = 56)	131.25 (63.9)
School year 7–9 (N = 130)	138.69 (71.9)
Bedtime/wake-up time	Mean (SD)
All students (N = 225)	22:03 (56 min)/6:29 (31 min)
School year 1–3 (N = 39)	21:08 (40 min)/6:20 (25 min)
School year 4–6 (N = 56)	21:42 (31 min)/6:32 (22 min)
School year 7–9 (N = 130)	22:30 (53 min)/6:32 (36 min)
Primary caregiver (N = 225)	22:49 (65 min)/5:43 (55 min)
SDQ	Mean (SD)
Total difficulties score	9.71 (5.21)
Conduct problem	1.58 (2.03)
Hyperactivity/inattention	2.97 (2.09)
Emotional symptoms	2.48 (2.35)
Peer problems	1.98 (1.74)
Prosocial behavior	6.56 (2.04)

Notes: Continuous variables are presented as mean (SD); categorical variables are presented as n (%). Abbreviations: min = minutes; SDQ = Strengths and Difficulties Questionnaire; SD = standard deviation.

**Table 3:** Stepwise multiple linear regression analysis for children’s screen time.

Variables	B	β	t	*p*	95% CI for B
Constant	144.588		10.671	<0.001	117.886 to 171.291
Children’s Sleep Duration	−19.908	−0.281	−4.528	<0.001	−28.573 to −11.243
Children’s Difficulty	2.159	0.166	2.680	0.008	0.572 to 3.747
Parental Control	−18.775	−0.204	−3.281	0.001	−30.053 to 3.747

Notes: Abbreviations: B = unstandardized coefficients B; β = standardized coefficients beta; CI = confidence interval. Adjusted R square = 0.142. The school year, considered a covariate to the sleep variable, was adjusted for “children’s sleep duration” in the factor analysis.

**Table 4:** Multiple mediation model: indirect effect of parental bedtime on children’s screen time.

			Product of Coefficients			Bootstrapping Bias-Correlated 95% CI
Relationship	B	β	SE	z	*p*	Lower to Upper
Parental Bedtime →Children’s sleep duration →Children’s screen time	5.926	0.095	1.982	2.988	0.001	2.744 to 10.833

Notes: Abbreviations: B = unstandardized coefficients; β = standardized coefficients; CI = confidence interval; SE = standard error. Bootstrapping sample size = 2000.

## Data Availability

The data presented in this study are available on request from the corresponding author. As no informed consent was given by the participants for open data sharing, not all data are freely accessible. However, the individual data are available upon reasonable request.

## References

[1] Brzęk A., Strauss M., Sanchis-Gomar F., Leischik R. (2021). Physical Activity, Screen Time, Sedentary and Sleeping Habits of Polish Preschoolers during the COVID-19 Pandemic and WHO’s Recommendations: An Observational Cohort Study. Int. J. Environ. Res. Public Health.

[2] Ministry of Education, Culture, Sports, Science, and Technology-Japan (2019). The Realization of the GIGA School Concept. https://www.mext.go.jp/a_menu/other/index_00001.htm.

[3] Schmidt M.E., Haines J., O’Brien A., McDonald J., Price S., Sherry B., Taveras E.M. (2012). Systematic review of effective strategies for reducing screen time among young children. Obesity.

[4] Wu L., Sun S., He Y., Jiang B. (2016). The effect of interventions targeting screen time reduction: A systematic review and meta-analysis. Medicine.

[5] Japan Pediatric Society (2004). Recommendations for “Children and Media” Issues. https://www.jpa-web.org/about/organization_chart/cm_committee.html.

[6] Robinson T.N., Banda J.A., Hale L., Lu A.S., Fleming-Milici F., Calvert S.L., Wartella E. (2017). Screen Media Exposure and Obesity in Children and Adolescents. Pediatrics.

[7] Hisler G.C., Hasler B.P., Franzen P.L., Clark D.B., Twenge J.M. (2020). Screen media use and sleep disturbance symptom severity in children. Sleep. Health.

[8] Song Y., Li L., Xu Y., Pan G., Tao F., Ren L. (2020). Associations between screen time, negative life events, and emotional and behavioral problems among Chinese children and adolescents. J. Affect. Disord..

[9] French A.N., Ashby R.S., Morgan I.G., Rose K.A. (2013). Time outdoors and the prevention of myopia. Exp. Eye Res..

[10] Sharif I., Sargent J.D. (2006). Association between television, movie, and video game exposure and school performance. Pediatrics.

[11] Novaković S., Milenković S., Srećković M., Backović D., Ignjatović V., Capo N., Stojanović T., Vukomanović V., Sekulić M., Gavrilović J. (2023). Children’s Internet use and physical and psychosocial development. Front. Public Health.

[12] Cartanyà-Hueso À., Lidón-Moyano C., Martín-Sánchez J.C., González-Marrón A., Matilla-Santander N., Miró Q., Martínez-Sánchez J.M. (2021). Association of screen time and sleep duration among Spanish 1–14 years old children. Paediatr. Perinat. Epidemiol..

[13] Lissak G. (2018). Adverse physiological and psychological effects of screen time on children and adolescents: Literature review and case study. Environ. Res..

[14] Parent J., Sanders W., Forehand R. (2016). Youth Screen Time and Behavioral Health Problems: The Role of Sleep Duration and Disturbances. J. Dev. Behav. Pediatr..

[15] Magee C.A., Lee J.K., Vella S.A. (2014). Bidirectional relationships between sleep duration and screen time in early childhood. JAMA Pediatr..

[16] Lo C.B., Waring M.E., Pagoto S.L., Lemon S.C. (2015). A television in the bedroom is associated with higher weekday screen time among youth with attention deficit hyperactivity disorder (ADD/ADHD). Prev. Med. Rep..

[17] Swing E.L., Gentile D.A., Anderson C.A., Walsh D.A. (2010). Television and video game exposure and the development of attention problems. Pediatrics.

[18] Takahashi N., Tsuchiya K.J., Okumura A., Harada T., Iwabuchi T., Rahman M.S., Kuwabara H., Nomura Y., Nishimura T. (2023). The association between screen time and genetic risks for neurodevelopmental disorders in children. Psychiatry Res..

[19] Cortese S., Wang F., Angriman M., Masi G., Bruni O. (2020). Sleep Disorders in Children and Adolescents with Autism Spectrum Disorder: Diagnosis, Epidemiology, and Management. CNS Drugs.

[20] Ahlberg R., Garcia-Argibay M., Taylor M., Lichtenstein P., D’Onofrio B.M., Butwicka A., Hill C., Cortese S., Larsson H., Du Rietz E. (2023). Prevalence of sleep disorder diagnoses and sleep medication prescriptions in individuals with ADHD across the lifespan: A Swedish nationwide register-based study. BMJ Ment. Health.

[21] Wu X., Tao S., Rutayisire E., Chen Y., Huang K., Tao F. (2017). The relationship between screen time, nighttime sleep duration, and behavioural problems in preschool children in China. Eur. Child. Adolesc. Psychiatry.

[22] Kim K.W., Koh Y.K., Kim J.H. (2022). Associations between Parental Factors and Children’s Screen Time During the COVID-19 Pandemic in South Korea. Child. Psychiatry Hum. Dev..

[23] Leonard H., Khurana A. (2022). Parenting Behaviors and Family Conflict as Predictors of Adolescent Sleep and Bedtime Media Use. J. Youth Adolesc..

[24] Jago R., Wood L., Zahra J., Thompson J.L., Sebire S.J. (2015). Parental control, nurturance, self-efficacy, and screen viewing among 5- to 6-year-old children: A cross-sectional mediation analysis to inform potential behavior change strategies. Child. Obes..

[25] Komada Y., Adachi N., Matsuura N., Mizuno K., Hirose K., Aritomi R., Shirakawa S. (2009). Irregular sleep habits of parents are associated with increased sleep problems and daytime sleepiness of children. Tohoku J. Exp. Med..

[26] Matsuishi T., Nagano M., Araki Y., Tanaka Y., Iwasaki M., Yamashita Y., Nagamitsu S., Iizuka C., Ohya T., Shibuya K. (2008). Scale properties of the Japanese version of the Strengths and Difficulties Questionnaire (SDQ): A study of infant and school children in community samples. Brain Dev..

[27] Cabinet Office, Government of Japan (2015). White Paper on Children and Youth. https://www8.cao.go.jp/youth/whitepaper/h27honpen/b1_06_01.html.

[28] NHK Broadcasting Culture Research Institute (2020). National Living Time Survey. https://www.nhk.or.jp/bunken/yoron-jikan/column/sleep-2020.html.

[29] Pan W., Jiang L., Geng M.L., Ding P., Wu X.Y., Tao F.B. (2019). Correlation between screen-watching time and emotional problems as well as combination effect of outdoor time among preschool children. Zhonghua Liu Xing Bing. Xue Za Zhi.

[30] Thomas M.M., Gugusheff J., Baldwin H.J., Gale J., Boylan S., Mihrshahi S. (2020). Healthy Lifestyle Behaviours Are Associated with Children’s Psychological Health: A Cross-Sectional Study. Int. J. Environ. Res. Public Health.

[31] Niiranen J., Kiviruusu O., Vornanen R., Saarenpää-Heikkilä O., Paavonen E.J. (2021). High-dose electronic media use in five-year-olds and its association with their psychosocial symptoms: A cohort study. BMJ Open.

[32] Khan A., Uddin R., Burton N.W. (2018). Insufficient physical activity in combination with high screen time is associated with adolescents’ psychosocial difficulties. Int. Health.

[33] Hale L., Guan S. (2015). Screen time and sleep among school-aged children and adolescents: A systematic literature review. Sleep. Med. Rev..

[34] Christensen M.A., Bettencourt L., Kaye L., Moturu S.T., Nguyen K.T., Olgin J.E., Pletcher M.J., Marcus G.M. (2016). Direct Measurements of Smartphone Screen-Time: Relationships with Demographics and Sleep. PLoS ONE.

[35] Guerrero M.D., Barnes J.D., Chaput J.P., Tremblay M.S. (2019). Screen time and problem behaviors in children: Exploring the mediating role of sleep duration. Int. J. Behav. Nutr. Phys. Act..

[36] Tambalis K.D., Panagiotakos D.B., Psarra G., Sidossis L.S. (2018). Insufficient Sleep Duration Is Associated with Dietary Habits, Screen Time, and Obesity in Children. J. Clin. Sleep. Med..

[37] Kahn M., Schnabel O., Gradisar M., Rozen G.S., Slone M., Atzaba-Poria N., Tikotzky L., Sadeh A. (2021). Sleep, screen time and behaviour problems in preschool children: An actigraphy study. Eur. Child. Adolesc. Psychiatry.

[38] Falbe J., Davison K.K., Franckle R.L., Ganter C., Gortmaker S.L., Smith L., Land T., Taveras E.M. (2015). Sleep duration, restfulness, and screens in the sleep environment. Pediatrics.

[39] Musa S., Elyamani R., Dergaa I. (2022). COVID-19 and screen-based sedentary behaviour: Systematic review of digital screen time and metabolic syndrome in adolescents. PLoS ONE.

[40] Ishtiaq A., Ashraf H., Iftikhar S., Baig-Ansari N. (2021). Parental perception on screen time and psychological distress among young children. J. Family Med. Prim. Care.

[41] Matsumoto Y., Kaneita Y., Jike M., Osaki Y. (2021). Clarifying the factors affecting the implementation of the “early to bed, early to rise, and don’t forget your breakfast” campaign aimed at adolescents in Japan. Sleep. Biol. Rhythms.

